# Unpacking the Complexities of a Silent Killer

**DOI:** 10.3390/ijms24087125

**Published:** 2023-04-12

**Authors:** Dai Yamanouchi

**Affiliations:** Division of Vascular Surgery, Department of Surgery, University of Wisconsin School of Medicine and Public Health, Madison, WI 53705, USA; yamano@surgery.wisc.edu; Tel.: +1-608-265-4420

An abdominal aortic aneurysm (AAA) is a life-threatening condition that affects millions of people worldwide. It remains hidden and unnoticed during most of its development but ultimately results in the patient’s death. AAA-related mortality remains significantly high, with its prevalence estimated to be around 1.1 million in the United States [1,2,3]. A ruptured AAA is responsible for causing the death of over 5000 people annually in the U.S., making it the 15th biggest killer overall and the 10th biggest killer for men aged 55 years and above [4]. Statistics reveal that 4% to 5% of sudden deaths are caused by a ruptured AAA [5,6]. Unfortunately, almost half of the patients with a ruptured AAA do not survive, and those who reach the hospital also have a low survival rate, with 80% to 90% mortality under all circumstances [7,8].

The early detection and proper management of AAAs is critical to preventing serious consequences. However, despite its devastating impact, it remains under-diagnosed and under-researched, largely due to its silent nature. The current diagnostic options for AAA are limited, with ultrasound being the most widely used method for detection. In many cases, an AAA is discovered accidentally, only when patients present with symptoms of rupture. This highlights the need for improved diagnostic methods for AAA, which could lead to earlier detection and improved outcomes for patients [7,8].

Advances in molecular research have shed new light on the underlying mechanisms of AAAs and opened up new avenues for diagnosis and treatment. In this Special Issue of the *International Journal of Molecular Sciences*, we bring together leading researchers from around the world to present the latest findings on AAAs. From exploring the genetics of AAAs to understanding the molecular basis of its progression, this issue provides a comprehensive overview of the current state of the field.

The authors in this Special Issue explore innovative approaches to addressing the challenges posed by AAAs. By incorporating cutting-edge technologies and innovative research methods, they are working towards a better understanding of the underlying mechanisms of AAAs and the development of new and more effective diagnostic and treatment options. Through their contributions, these authors are leading the way in the fight against AAAs and providing hope for those affected by this devastating condition.

The authors in this Special Issue present state-of-the-art approaches to addressing the challenges posed by AAAs. For example, Fukuhara et al. developed a polymeric micelle loaded with a statin drug for treating AAAs in rat models. Polymeric micelles are nanomedicines that selectively deliver drugs to disease sites, potentially improving the therapeutic efficacy. The study found that micelles prevented aortic aneurysm expansion in a dose-dependent manner and decreased macrophage infiltration and matrix metalloproteinase-9 (MMP-9) activity in cases of an AAA. This research presents a promising approach to developing systemically injectable drugs for treating AAAs with improved bioavailability [9].

Yoshimura et al. developed a novel drug delivery system for inhibiting aortic wall degeneration and aneurysm exclusion failure after endovascular aneurysm repair with a stent graft. The system involved a targeted graft labeled with a small target molecule and a target-recognizing nanocarrier containing suitable drugs. The researchers successfully demonstrated that the nanocarriers could bind to the targeted graft both in vitro and in blood vessels of live mice. The drug released from the system reduced the expression of MMP-9 in mouse aortas, representing a promising adjuvant therapy to improve the long-term outcomes of endovascular aneurysm repair [10].

Plana et al. investigated dysregulated microRNAs (miRNAs) in plasma and tissue samples of patients with an AAA. The study found that miR-27b-3p and miR-221-3p were overexpressed in the plasma of patients with an AAA, while six miRNAs were underexpressed and four miRNAs were overexpressed in AAA tissue. Thrombospondin-2 was identified as a potential target of miR-195-5p in AAA tissue. These findings suggest that miRNAs may play a role in AAA pathogenesis and could be potential therapeutic targets [11].

Hayashi-Hori et al. investigated the effectiveness of rapamycin, an mTOR inhibitor, in preventing and suppressing aortic dissection (AD) in a mouse model. Rapamycin was found to be effective in preventing AD development and suppressing its progression by suppressing cell cycle-related genes, inducing muscle development-related genes, and maintaining the contractile phenotype of aortic smooth muscle cells. These findings suggest that the mTOR pathway plays a crucial role in AD pathogenesis and that rapamycin may be a potential treatment option for AD in clinical practice [12].

Krishna et al. reviewed the current literature on mouse models of AAAs to study the factors that influence AAA rupture, such as the aortic wall structure and strength, biomechanical forces, and the cellular and proteolytic composition of the AAA wall. AAA rupture, which is a major cause of death in older adults, occurs when the stress on the aneurysm wall exceeds the wall strength. Mouse models offer a unique opportunity to study these factors and develop surrogate markers of AAA rupture in patients. However, the review also highlights the limitations of mouse models and suggests innovative approaches to generate clinically relevant results in future experimental AAA studies [13].

Gurung et al. analyzed the current literature on the genetic and epigenetic mechanisms involved in the role of smooth muscle cells (SMCs) in AAA formation. An AAA is characterized by thinning of the media and adventitia of the aortic wall, which leads to aortic rupture and high mortality if left untreated. This review highlighted the importance of SMC plasticity, which is characterized by phenotypic modulation towards de-differentiation and a proliferative state that is associated with extracellular matrix remodeling, cell senescence, and inflammation in the pathogenesis of AAAs. While the evidence from mouse models is convincing, the authors emphasized the urgent need to apply this knowledge to human biology using modern experimental technology [14].

In addition to the latest findings in molecular research on AAAs presented in this Special Issue, our understanding of the pathophysiology of AAAs continues to expand. Aneurysmal degeneration of the aortic wall is caused by the degeneration of elastin in the aortic wall [8]. Our laboratory and others have demonstrated that medial accumulation of macrophages in mouse AAA models and human aneurysms are a hallmark of disease progression [15,16,17,18,19]. It has been shown that the macrophages accumulating in AAAs derive mostly from circulating monocytes, which are produced in the bone marrow and can be mobilized from peripheral reservoirs such as the spleen [20,21]. Moehle et al. highlighted the importance of macrophages by demonstrating that deleting MCP-1 in bone marrow-derived cells abrogates AAA formation [20]. The three most commonly used mouse AAA models are adventitial exposure to CaCl2, transient perfusion of elastase into the infrarenal aorta, and chronic subcutaneous infusion of angiotensin II [22]. We and others have demonstrated that macrophage-derived MMP-9 involvement in aneurysm formation is a characteristic of all three models [8,15,19,23,24,25]. We have also identified a unique population of macrophages within human and mouse AAA tissue that express markers of osteoclasts (OC) [15,24,26,27].

Previous research from our group has established that cigarette smoking is a significant risk factor for AAAs, and a better understanding of the effect of cigarette smoke on AAAs has the potential to reveal new therapeutic targets [1]. Our group has focused on the dysregulation of macrophage proteases in AAA and found a unique macrophage population that shares similar characteristics with bone OC [24]. These OC-like (OCL) macrophages demonstrate binding of receptor activator of NF-kappaB ligand (RANKL) to its receptor, RANK, which is best known as the key stimulus for OC differentiation. In AAAs, we found that approximately 50% of macrophages form RANKL–RANK complexes on the cell surface that trigger activation of nuclear factor of activated T cells c1 (NFATc1) and expression of OC-related genes including MMP-9. Previous research in a mouse model has shown that cigarette smoke extract (CSE) treatment significantly exacerbates OCL macrophages and AAA formation, accompanied by upregulation of hypoxia-inducible factor-1 alpha [28]. 

This Special Issue of the *International Journal of Molecular Sciences* thus provides a comprehensive overview of the current state of molecular research on AAAs, highlighting the importance of continued investigation into the underlying mechanisms of this life-threatening condition. From exploring the genetics of AAAs to understanding the molecular basis of its progression, this issue demonstrates the potential for further advancements in the diagnosis and treatment of AAAs. The collection of ground-breaking research presented in this issue provides a valuable resource for researchers, clinicians, and other professionals working in the field of AAAs. It also provides insights into the future directions of the field, inspiring further research and collaboration towards better outcomes for patients with AAAs.
